# Special Issue: Childhood Brain Cancer Treatment

**DOI:** 10.3390/cancers15215278

**Published:** 2023-11-03

**Authors:** Stefano Mastrangelo

**Affiliations:** Pediatric Oncology Unit, Fondazione Policlinico Universitario A. Gemelli—IRCCS, Università Cattolica del Sacro Cuore di Roma, Largo A. Gemelli, 8, 00168 Rome, Italy; stefano.mastrangelo@unicatt.it; Tel.: +39-30-155-165

Brain cancer is the second most common childhood malignancy and is the leading cause of death among all pediatric cancers [1]. In recent decades, there has been an improvement in neuroimaging, surgical technics, radiotherapy, and chemotherapy, with an increase in survival rates in children with medulloblastoma and low-grade glioma [2], while for other tumors, such as high-grade gliomas [3,4] or recurrent malignant tumors, it remains dismal [5]. With the more recent use of high-resolution epigenetic and genomic profiling, important advances have been achieved in our understanding of the molecular alterations of almost every type of pediatric brain tumor. This information has also helped to identify and categorize tumors into different subsets, recognized by the 2021 fifth edition World Health Organization classification of central nervous system tumors [6]. Moreover, the basis for risk-adapted treatment stratification has been formed, and new classes of molecularly targeted therapeutic agents have been revealed, which are especially needed for pediatric brain tumors with poor prognosis.

Two papers of this Special Issue highlight aspects of radiation therapy, the most antineoplastic tool for pediatric brain tumors after surgery, regarding the long-term cognitive sequelae and the possible use of innovative helium ions in particular. Other papers of this Special Issue focus on two of the most aggressive pediatric brain tumors, which bear very different prognoses: medulloblastoma, for which, with current treatment protocols, a good long-term prognosis can be achieved, and H3.3K27M mutated diffuse midline glioma, which carries one of the worst prognoses of all pediatric brain tumors. Finally, a review paper analyzes two cellular pathways involved in tumoral proliferation, differentiation, migration, and angiogenesis, and current targeted therapies available for a new approach against brain tumors, opening the possibility for future scenarios of cure even for the most lethal high-grade histologies.

Surgery still represents the most effective therapeutic tool for all types of brain tumors, and a complete or gross total resection is correlated with a better prognosis. Nevertheless, only low-grade gliomas maintain an excellent long-term prognosis without further treatment, while for other tumor histologies, in particular high-grade gliomas and ependymomas, radiotherapy (RT) is essential to prevent local recurrence or to reduce residual tumors. After irradiation of patients with pediatric brain tumors, late sequelae, such as neurocognitive decline, limit the doses that can be delivered, especially in younger children. More recently, advanced radiation techniques with increased conformality have improved cognitive outcomes. Weusthof et al. (contribution 1) reported data of a study based on 103 children (aged 1.6 to 19 years) with brain tumors, in which neurocognitive outcomes were evaluated before and after treatment, and for up to four years of follow-up. The different treatment modalities were compared through longitudinal assessment of seven neurocognitive domains, in adjunction to the evaluation of the overall effect on scholastic performance. After both photon RT and surgery, a decline in processing speed, non-verbal intelligence, and visuospatial abilities was observed. In contrast, following proton RT, no alterations in long-term neurocognitive abilities were reported. These results suggested that modern radiation therapy affects neurocognition much less than in the past. Moreover, proton therapy has been demonstrated to increase target coverage and reduce organ damage in comparison to conventional photon radiation. In another paper of this Special Issue, Wickert et al. (contribution 2) analyzed the data of 15 pediatric ependymoma patients who were treated with adjuvant proton therapy and also evaluated them with calculated plans for both innovative helium ion treatment and the highly conformal IMRT to compare the different treatments. It was confirmed that the dose absorbed by healthy brain tissue could be significantly reduced with protons by up to −48% vs. IMRT photon radiotherapy. Moreover, they demonstrated that helium ions had the potential to reduce the absorbed dose for critical neuronal structures by up to another −39% despite similar target coverage. The authors provide a strong rationale for the use of helium ions due to their potential to further reduce the risk of long-term sequelae following radiation therapy.

The paper by Fang et al. (contribution 3) is a review on medulloblastoma, which represents one third of all pediatric brain tumors, thus being the most frequent histology. Four subtypes of medulloblastoma (WNT, Sonic hedgehog (SHH), Group 3, and Group 4) are described according to the 2016 WHO classification, which precede the modified subtypes described in the new 2021 classification. Tumor analysis and clinical management of medulloblastoma with a focused discussion on the most recent developments of imaging, surgical, radiotherapeutic, and chemotherapeutic intervention are described, including epigenetics and possible therapeutic targeting. In their research paper, Rohere et al. (contribution 4) investigated the role of activated STAT3 in promoting medulloblastoma pathogenesis and chemoresistance. In their study, survival, proliferation, anti-apoptosis, migration, expression of MYC, and stemness, which determine the tumorigenicity of medulloblastoma cells, were reduced by targeting STAT3 function. The authors showed that in subcutaneous and intracranial orthotopic xenografts, medulloblastoma tumor growth was reduced and the sensitivity to cisplatin increased. Therefore, it is postulated that, in the future, STAT3 targeting could be an adjuvant therapy and chemo-sensitizer to increase the efficacy of treatment, with the aim of reducing treatment toxicity and improving quality of life.

As prognosis for tumors such as high-grade gliomas and, in particular, diffuse intrinsic pontine glioma continues to be dismal, it is of fundamental importance to improve the characterization of these tumor types to achieve a risk-adapted treatment stratification and eventually increase cure rates through the use of novel treatment agents. In the review paper by Argersinger et al. (contribution 5), H3K27M-mutant diffuse midline glioma is described. This rare pediatric brain tumor that originates in midline brain structures has a very poor prognosis with the available therapies, and therefore new and better treatment agents are required. The authors describe the clinical characteristics, diagnostic methods, and pathogenesis of this type of tumor and then focus on molecular tumor characteristics and relative therapeutic targets. Preclinical studies and clinical trials utilizing immunotherapy, radiation, and chemotherapy against this cancer are summarized, and recent advances in immunotherapy and molecular biology are described. Also in this Special Issue, Rakotomalala et al. (contribution 6) have published a study regarding H3.3K27M mutations in diffuse midline glioma and its resistance to both radio- and chemotherapy, which determines the dismal prognosis. They observed that the H3.3K27M mutation triggered an increase in cell growth in pediatric glioma cell line Res259 and SF188 cells, associated with higher clonogenic capacities, and also modulated the response of Res259 cells to different classes of treatment compounds. Moreover, it was shown that the mutation confers an increased resistance to ionizing radiations in Res259 and KNS42 cells. Indeed, the authors demonstrated that H3.3K27M can increase cell radioresistance capabilities independently of TP53 alterations. The data presented in this paper confirm the tumorigenic role of H3.3K27M mutation and highlight its strong involvement in resistance to therapies in pediatric glioma cells.

As mentioned, the future of pediatric brain tumor treatment also relies on the identification of specific molecular alterations and the development of new targeted therapies. In the paper by Talloa et al. (contribution 7), the mechanisms of two cellular pathways are reviewed: RAS/RAF/MEK/MAPK and PI3K/AKT/mTOR, which are involved in tumoral proliferation, differentiation, migration, and angiogenesis. In particular, the BRAF mutation, a component of both the MAPK and PI3K/AKT/mTOR pathways, is analyzed. In the second part of the paper, data are reported regarding current targeted therapies with BRAF and MEK inhibitors used in pediatric low-grade gliomas, high-grade gliomas, and other central nervous system tumors that often present these mutations.

In conclusion, this Special Issue highlights recent advances in radiation therapy for pediatric brain tumors and focuses on medulloblastoma, the most common histologic tumor type, and on diffuse midline glioma with H3.3K27M mutation, the most lethal type. Molecular analysis and target therapies are also described, with the hope that they can lead to a further improvement in the long-term prognosis of pediatric malignant brain tumors.
